# Is it Time for Reviewer 3 to Request Human Organ Chip Experiments Instead of Animal Validation Studies?

**DOI:** 10.1002/advs.202002030

**Published:** 2020-10-12

**Authors:** Donald E. Ingber

**Affiliations:** ^1^ Wyss Institute for Biologically Inspired Engineering at Harvard University Boston MA 02115 USA; ^2^ Vascular Biology Program, Department of Surgery Boston Children's Hospital and Harvard Medical School Boston MA 02115 USA; ^3^ Harvard John A. Paulson School of Engineering and Applied Sciences Cambridge MA 02138 USA

**Keywords:** microfluidics, microphysiological systems, organoids, organ‐on‐a‐chip, preclinical studies

## Abstract

For the past century, experimental data obtained from animal studies have been required by reviewers of scientific articles and grant applications to validate the physiological relevance of in vitro results. At the same time, pharmaceutical researchers and regulatory agencies recognize that results from preclinical animal models frequently fail to predict drug responses in humans. This *Progress Report* reviews recent advances in human organ‐on‐a‐chip (Organ Chip) microfluidic culture technology, both with single Organ Chips and fluidically coupled human “Body‐on‐Chips” platforms, which demonstrate their ability to recapitulate human physiology and disease states, as well as human patient responses to clinically relevant drug pharmacokinetic exposures, with higher fidelity than other in vitro models or animal studies. These findings raise the question of whether continuing to require results of animal testing for publication or grant funding still makes scientific or ethical sense, and if more physiologically relevant human Organ Chip models might better serve this purpose. This issue is addressed in this article in context of the history of the field, and advantages and disadvantages of Organ Chip approaches versus animal models are discussed that should be considered by the wider research community.

## Graphical abstract

This article reviews recent advances with human Organ Chip microfluidic culture technology showing that it can mimic human physiology, disease states, and responses to drugs with higher accuracy than some animal models. This raises the question whether it might be time for reviewers of publications and grants to consider requesting human Organ Chip studies in lieu of animal testing.

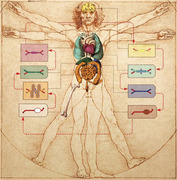

## 1 Introduction

Biomedical research grant applications and manuscripts commonly begin with an explanation of the potential clinical relevance of the work for human health. Most basic researchers carry out experiments using in vitro models, and thus, key to their premise is that the results they generate in their cell cultures will translate to humans. Because in vitro studies generally lack the natural three dimensional (3D) context, vascular flow, and physico‐chemical microenvironment of living tissues and organs, as well as the multi‐organ physiology of whole organisms, many question the clinical relevance of findings obtained with these simplified models. For this reason, most researchers who submit a grant application or publication based on in vitro findings commonly expect to find at least one reviewer (the classic exasperating “Reviewer 3”) who demands that additional animal experiments be carried out to validate their findings before the work could be acceptable for publication or funding. This article seeks to provoke a conversation in the scientific community by asking two simple questions: does this make sense, and if not, is there a better alternative? I address these questions by reviewing recent progress that has been made using organoids and engineered microphysiological systems (MPS) with a focus on microfluidic organ‐on‐a‐chip (Organ Chip) culture technologies.

## 2 Why Do We Require Animal Studies to Validate In Vitro Findings?

Animal experiments have been the mainstay of scientific research since the time of Aristotle, and there are good reasons for this. Many molecular mechanisms, physiological processes, and disease states involve complex tissue‐ and organ‐level structures and functions, as well as interplay among multiple organ systems. The effects that drugs produce in our bodies not only depend on their effects within individual cells; they are also governed by their pharmacokinetics (PK), which defines how their levels change over time in flowing blood and in different tissues depending on the dynamics of their absorption, distribution, metabolism, and excretion (ADME) behaviors. It is not possible to model these complex responses, disease phenotypes, and PK behaviors in vitro using conventional culture technology, and hence, it has been difficult to study the relation between molecular mechanism, pathogenesis, and effects of drugs or toxins in a meaningful way without carrying out animal experiments. Another excellent reason for doing animal studies is when experiments in humans would be unethical, for example, when testing the effects of exposure to lethal toxins, high energy radiation, or potentially deadly pathogens, such as infection by Ebola or the SARS‐CoV‐2 virus.

Reviewers of grants and publications appreciate the importance of animal models for all of the above reasons, but perhaps more central to their argument is the ability of animals, and particularly mice, to be genetically engineered to mimic human disease states. This has been one of the central tenets of basic biomedical research for the past 40 years, particularly in studies focused on discovery of new therapeutics to alleviate human suffering. But are animal models good predictors of human patient outcomes? The answer to this question might be surprising.

Studies by the pharmaceutical industry, as well as regulatory and government agencies around the world, including the United States Food and Drug Administration (FDA) and National Institutes of Health (NIH), have consistently found that results from drug testing in preclinical animal models are poor predictors of human responses.^[^ ^1^, ^2^, ^3^ ^]^ Estimates of failure rates range as high 90%, and this relates to both lack of efficacy and safety problems when the drugs are evaluated in human clinical trials.

Many animal models, including genetically engineered mice, can mimic human disease phenotypes; however, the underlying molecular, cellular, and physiological mechanisms are often distinct. For example, while mice can generate phenotypes reminiscent of multi‐system inflammatory responses in humans, such as sepsis and acute respiratory distress (ARDS), the genomic pathways that underlie these responses differ significantly.^[^ ^4^ ^]^ Similarly, while mice can be engineered to develop neurofibrillary tangles similar to those seen in the brains of humans with Alzheimer's Disease, the molecular mechanisms uncovered in mouse models and drugs developed based on these insights have not translated well when the drugs were moved into the clinic.^[^ ^5^ ^]^

Perhaps this is why hundreds of drugs that were found to be effective when tested on animals for diseases ranging from stroke to inflammation were not found to be active in humans, and why many vaccines have similarly failed in clinical trials even after working well in non‐human primates.^[^ ^6^ ^]^ Furthermore, recent advances in the biotechnology industry have led to biologic therapies, such as therapeutic monoclonal antibodies, that are so specific for their human targets that they do not even cross react with related molecules in non‐human primates, and so there is no other option than to test them in a human model. Finally, a major issue that has always concerned the field of drug development is that there may be many therapies out there that were killed because they failed to produce positive results in animals, yet they could have potentially saved human lives. So why is Reviewer 3 still demanding that animal studies be carried out before scientific findings can be shared openly with the wider community or funding is provided to advance a potentially important research program? It is likely because they feel that there is no alternative; but this is not the case.

## 3 Are there Viable In Vitro Alternatives to Animal Models?

### 3.1 Human Organoids

**Figure 1:**
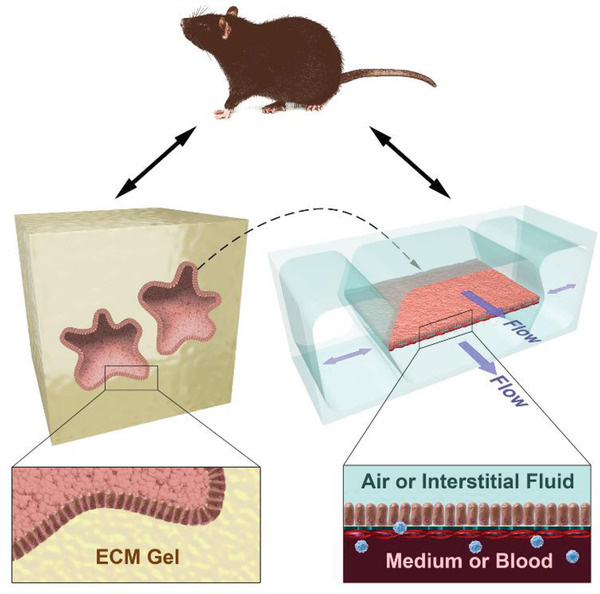
Potential in vitro replacements for animal testing. Schematics of mice, organoids that grow as small closed spheres which undergo organ‐specific cyto‐ and histo‐differentiation within 3D ECM gels, and microfluidic Organ Chips that may be lined with cells from organoids (dashed line), iPS cell‐derived cells, or primary cells. Organ Chips reconstitute tissue–tissue interfaces between organ‐specific epithelium and endothelium, vascular perfusion, interstitial flow or air–liquid interfaces, and mechanical cues (e.g., by applying cyclic strain via application of suction to hollow side chambers) to mimic breathing or peristalsis motions. Circulating or resident immune cells, connective tissue cells, nerve cells, and other cell types can be integrated into the Organ Chips as needed to recapitulate increasing levels of complexity.

Over the past decade, there have been many advances in culture technology that provide levels of cytodifferentiation, tissue functionalities, and organ‐level responses not possible in the past. Organoids, for example, are clusters of cells that contain a subset of self‐renewing stem cells, which grow and self‐organize into small closed spheres that undergo cytodifferentiation and recreate 3D tissue‐like structures and functions seen in living organs when grown embedded within solid extracellular matrix (ECM) gels in vitro (**Figure** **1**).^[^ ^7^ ^]^ Methods for generating organoids have been published for many organs, including intestine, lung, liver, kidney, and brain, among others, and because they contain stem cells, they can be used to continuously expand organ‐specific human cell populations for additional in vitro studies.

Importantly, organoids generated with human patient‐derived cells or human‐induced pluripotent stem (iPS) cells have proven to be extremely valuable for studying mechanisms relating to differentiation, morphogenesis, pathogenesis, and drug action, as well as discovery of disease biomarkers that act at the cell and tissue levels.^[^ ^7^ ^]^ Thus, they have a key role to play in the preclinical drug development pipeline, and they may be used to replace animal studies that are focused on cell and molecular mechanisms of disease.

However, because organoids are closed structures that lack tissue–tissue interfaces, vascular flow, circulating immune cells, and physiologically relevant mechanical cues, they cannot fully recapitulate organ‐level responses or be used to study drug effects under pharmacologically relevant conditions that depend upon dynamic drug PK exposure profiles. Integration of self‐organized capillary networks within 3D organoid cultures^[^ ^8^ ^]^ and addition of immune cells to the surrounding ECM gel^[^ ^9^ ^]^ may help to overcome some of these limitations. But there are still significant limitations, such as an inability control vascular architecture and flow dynamics, and hence how chemicals, inflammatory molecules, drugs, and immune cells are transported to and from these tissues as they are within living vascularized organs in our bodies. Because organoids are closed structures surrounded by a thick dense ECM, it is also difficult to measure of transport and absorption of nutrients, chemicals, or drugs across the epithelial tissue, experimentally sample contents of the internal lumen, sustain co‐cultures with living microbiome, and integrate sensors for on‐line functional measurement using these models.

### 3.2 Static Microphysiological Systems

Over the past decade, there also has been an explosion in the development of advanced MPS, such as engineered 3D tissues and microfluidic Organ Chips with dynamic flow. Some static MPS models that lack fluid flow can produce high levels of functionality and mimicry of tissue functions, and they have been used in combination with primary human cells or iPS‐derived cells from patients to gain insight into human physiology and pathogenesis that would be difficult to accomplish using animal models. For example, contractile heart tissues engineered using iPS cell‐derived cardiomyocytes from patients with Barth syndrome were able to mimic key features of the cardiomyopathy these patients experience in vitro, and this work led to new mechanistic insights into the pathogenesis of this disease.^[^ ^10^ ^]^ Tissue engineering with iPS‐derived cardiomyocytes from human patients along with physiological electrical conditioning also enabled development of electrophysiologically distinct atrial and ventricular tissues that mimicked chamber‐specific drug responses and gene expression, which permitted modeling of polygenic left ventricular hypertrophy in vitro.^[^ ^11^ ^]^ There are also numerous other examples of how static MPS models can mimic human cell and tissue pathophysiology, as well as cellular responses to drug therapies, and so they too represent viable replacements for some animal studies.^[^ ^12^ ^]^ However, these MPS models do not enable dynamic flow required to mimic drug PK or support multi‐organ coupling, and hence, they only satisfy a subset of requirements for replacing animal studies, much like organoids.

### 3.3 Microfluidic Organ Chips

Microfluidic Organ Chips are a form of dynamic MPS that recapitulate vascular perfusion, tissue–tissue interfaces, and organ‐relevant mechanical motions, while also allowing integration of circulating immune cells, connective tissues cells, and complex microbiome, which could serve as meaningful alternatives to many types of animal testing (Figure 1). These devices may be lined with either primary cells, iPS cells, or cells isolated from patient‐derived organoids. Importantly, while organoids may be maintained in perfusion cultures, the advantage of microfluidic Organ Chips is that dynamic fluid flow is delivered through an endothelium‐lined channel as it is in living organs, and hence nutrients, wastes, and drugs are transferred back and forth across the endothelial interface with surrounding tissues in the chip as occurs in vivo.

Many Organ Chip designs have been explored,^[^ ^13^ ^]^ however, a common embodiment is a small microfluidic device the size of a computer memory stick composed of an optically clear flexible polymer, such as poly‐dimethylsiloxane (PDMS), that contains two linear hollow channels each less than 1 mm wide that are separated by a thin porous membrane made of the same clear flexible material (Figure 1).^[^ ^14^ ^]^ By coating the membrane with ECM, it is possible to culture and differentiate organ‐specific epithelial cells on one side of the membrane while growing endothelium from the same organ on the other. Culture medium or whole blood can be flowed through the endothelium‐lined microchannel to mimic vascular perfusion, and medium can be flowed through the epithelial channel as a form of interstitial fluid (e.g., in Liver Chips) or the channel may be filled with air to create an air–liquid interface (ALI) (e.g., in Lung Chips). Immune cells, either circulating (Figure 1) or resident, as well as connective tissue cells (e.g., fibroblasts, astrocytes), may be incorporated into these devices as well. In addition, by applying cyclic suction to full height side chambers on the either side of the central microchannels in some devices, it is possible to rhythmically stretch and relax the side walls and attached porous membrane with the adherent tissue–tissue interface (Figure 1), thereby mimicking organ‐relevant motions, such as breathing in the lung or peristalsis in the intestine.

Importantly, many different Organ Chips have been shown to recapitulate human physiology, disease states, host–microbiome interactions, and responses to clinically relevant drug and radiation exposures with a level of fidelity that is as good or better than animal models. For example, a Lung Alveolus Chip lined by human A549 lung alveolar epithelial cells and pulmonary vascular endothelium that experienced cyclic breathing movements reproduced many features of human lung physiology including gas exchange and responses to bacteria, inflammatory cytokines, and airborne nanoparticulates introduced into the alveolar space, as well as revealing previously unknown effects of physiological breathing on these responses.^[^ ^14^ ^]^ The same chip mimicked drug toxicity‐induced pulmonary edema observed in human patients treated with the cancer drug interleukin‐2 (IL‐2) at a clinically relevant dose and over the same time frame, in addition to revealing that physiological breathing motions were required for this response while immune cells were not.^[^ ^15^ ^]^ Importantly, this work led to the identification of a potential therapeutic (TRPV4 ion channel inhibitor) and helped to enable its entry into human clinical trials.

A later version of the Lung Alveolus Chip lined with primary human lung alveolar cells and pulmonary microvascular endothelium that supported flowing whole human blood through the device faithfully recapitulated organ‐level contributions to platelet–endothelial dynamics and inflammation‐induced thrombosis.^[^ ^16^ ^]^ Using this chip, it was discovered that bacterial lipopolysaccharide (LPS) endotoxin stimulates intravascular thrombosis via activation of the alveolar epithelium, rather than acting directly on endothelium. In addition, this device was leveraged to deliver a potential adenoviral vector‐based gene therapy encoding a TRPV4 inhibiting peptide directly to the lung alveolar cells (i.e., through the airspace of the epithelial channel) while experience breathing motions, and this was able to suppress pulmonary vascular leakage.^[^ ^17^ ^]^ Thus, in addition to mimicking human responses to pathogens, toxins, drugs, and gene therapies, these Organ Chip studies led to new insights into the cellular and molecular basis of pathophysiology due to their ability to enable independent control of cell composition, chemical factors, fluid flow, and mechanical forces, which is not possible using either conventional cell culture, organoids, or animal models.

Human Lung Airway Chips lined by primary human bronchiolar epithelium grown under an ALI in one microchannel with an underlying microvascular endothelium in another also can reconstitute the goblet cell hyperplasia, increased cytokine production, and suppressed ciliary function of asthmatics when stimulated with interleukin‐13.^[^ ^18^ ^]^ When these same Airway Chips were lined with epithelial cells from patients with chronic obstructive pulmonary disease (COPD), they recapitulated the selective cytokine hypersecretion and enhanced recruitment of circulating human neutrophils that are flowed through the endothelium‐lined vascular channel, as well as clinical exacerbation by exposure to viral and bacterial toxins, which are seen clinically. This inflammatory disease model was also used to detect synergistic effects of lung endothelium and epithelium on cytokine production, identify potential biomarkers of COPD exacerbation, and measure responses to anti‐inflammatory drugs that suppress cytokine‐induced recruitment of circulating immune cells. Because the model incorporates dynamic fluid flow, it was possible to mimic recruitment of circulating immune cells and their inhibition by anti‐inflammatory drugs more effectively than static Transwell cultures that contained the same tissue–tissue interface.

**Figure 2:**
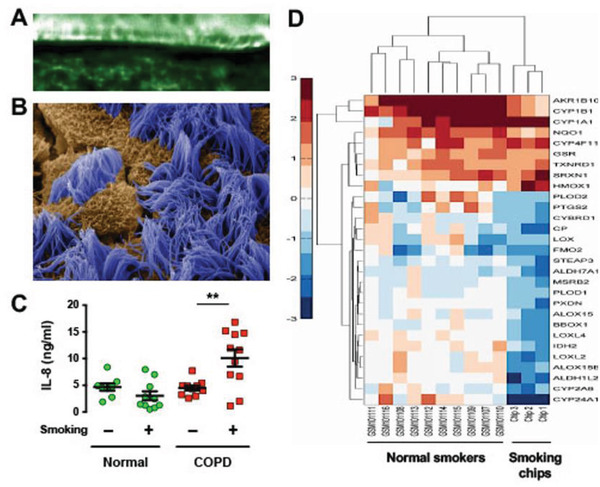
Human Lung Airway Chip recreates airway epithelial architecture and replicates patient responses to cigarette smoke in vitro. A) Still image captured from a video recording from the side of cilia beating on the apical surface of the differentiated airway epithelium on‐chip. B) Scanning electron micrograph of the apical surface of the airway epithelium formed on‐chip showing ciliated cells (blue) and non‐ciliated cells (brown). C) Graph showing changes in interleukin 8 (IL‐8) secretion in Lung Airway Chips lined with epithelial cells isolated from normal or COPD patients, with or without exposure to whole cigarette smoke for 75 min (smoking) (**p < 0.01). D) Heatmap comparing expression of 29 genes associated with cellular oxidation–reduction in lung airway epithelial cells obtained from small airways of different normal human smokers compared with samples obtained from three Lung Airway Chips that were exposed to whole cigarette smoke on‐chip. Note that the patterns of induced and suppressed genes in the Airway Chip mimic those seen in human patients. (B) Reproduced with permission.^[^ ^18^ ^]^ Copyright 2016, Springer Nature. (C,D) Reproduced with permission.^[^ ^19^ ^]^ Copyright 2016, Cell Press.

Furthermore, by connecting the Lung Airway Chips lined by primary human epithelial cells to a cigarette smoking robot that breathes whole cigarette smoke in and out of the air channel of the chips, it was possible to carry out matched comparative analysis of the same patients before and after smoke exposure. This led to the identification of ciliary abnormalities, as well as COPD‐specific cytokine and transcriptomic signatures (**Figure** **2**), that closely matched those detected in prior clinical studies in smokers who were otherwise healthy.^[^ ^19^ ^]^ Given the anatomic and physiological differences between species, these studies could not be done as effectively in animal models, and particularly not in mice given their small size.

Most recently, an influenza virus infection model was developed using the Lung Airway Chip, which replicates the virulence of different viral strains (e.g., H1N1 vs H3N2 and H5N1) and enables quantification of host cytokine and immune cell responses, as well as mimicry of clinical responses to antiviral therapies and spontaneous viral evolution in response to drug exposure that is enabled by human‐to‐human transmission.^[^ ^20^, ^21^ ^]^ Microfluidic Airway Chips are now being used to model human lung infection by SARS‐CoV‐2 virus in vitro, and to repurpose existing FDA‐approved drugs as potential COVID‐19 therapeutics.^[^ ^21^, ^22^ ^]^ Importantly, it is extremely difficult to develop animal models of viral infection, and none replicate human host responses as well as these Organ Chip models that incorporate highly differentiated human lung cells grown under an ALI while exposed to dynamic fluid flow at their base.

**Figure 3:**
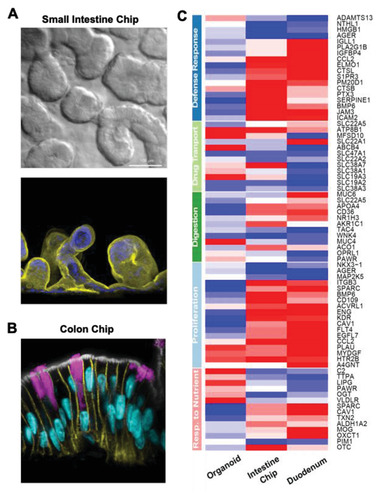
Human Intestine Chips lined by cells isolated from patient‐derived organoids exhibit differentiated structures and functions that closely resemble those displayed by living intestine in vivo. A) Differential interference microscopic image (top) and immunofluorescence microscopic image of F‐actin staining in green (bottom) of vertical sections through an Intestine Chip showing villus protrusions formed by primary intestinal (duodenal) epithelium cultured on‐chip for 12 days. B) Immunofluorescence microscopic image of a vertical section through a human Colon Chip showing a high polarized epithelium with basolateral adherens junctions labeled with E‐cadherin (green) and brush border stained for F‐actin (white) restricted to the apical regions, Hoechst stained nuclei (blue) localized at the cell base, and goblet cells stained for Muc2 (magenta). C) A curated heatmap showing gene expression profiles in the mechanically active Intestine Chip (with fluid flow and cyclic stretching to mimic peristalsis‐like motions) lined by cells from patient‐derived duodenal organoids, the duodenal organoids from which the cells were derived, and duodenum in vivo. Note that the expression of pattern of the Intestine Chip more closely resembles native intestine compared to the organoids. (A,C) Reproduced with permission.^[^ ^26^ ^]^ Copyright 2018, Springer Nature. (B) Reproduced with permission.^[^ ^27^ ^]^ Copyright 2020, Elsevier Inc.

The same chip design has been used to model the responses of human intestine to various stimuli in ways that are comparable to, or more accurate than, animal models. Intestine Chips that were initially lined by human Caco‐2 intestinal epithelial cells,^[^ ^23^, ^24^, ^25^ ^]^ and later by primary cells isolated from human patient‐derived duodenal or colon organoids along with primary human intestinal microvascular endothelium,^[^ ^26^, ^27^ ^]^ have been shown to exhibit histological appearance similar to that observed in vivo (e.g., villi‐like structures in duodenum) (**Figure** **3**A) and high levels of goblet cell differentiation (Figure 3B), as well as improved intestinal barrier function, enhanced drug metabolizing activities, and more mucus production than static cultures. Interestingly, when transcriptomic analysis was carried put, the Small Intestine Chips more closely resembled living duodenum in vivo than the organoids from which they were derived (Figure 3C).^[^ ^26^ ^]^ Moreover, the Colon Chips lined by organoid‐derived cells spontaneously accumulate a mucus layer with the same impenetrable and penetrable layers, and the same thickness, as is observed in human colon, whereas this is not observed in the organoids cultured under the same conditions without flow.^[^ ^27^ ^]^ Mucus accumulation in the Colon Chips also can be studied non‐invasively over time on‐chip using live imaging, which is not possible in other cell cultures or animal models.

Small Intestine Chips lined by cells isolated from patient‐derived organoids have been used to study induction of CYP450 metabolizing enzymes that can complicate the development of new drugs, and provide insight into the molecular and cellular basis of drug–drug interactions that can cause changes in drug PK, safety, and efficacy.^[^ ^28^ ^]^ Relative to animal models, Small Intestine Chips better mimic infection by enteric viruses^[^ ^29^ ^]^ and intestinal injuries induced by acute exposure to *γ*‐radiation (including dosage sensitivities and responses to countermeasure therapies) exhibited by humans as well.^[^ ^30^ ^]^

Additional Small Intestine Chip studies showed that probiotic and antibiotic therapies can prevent villus injury induced by pathogenic bacteria, and this led to identification of four proinflammatory cytokines (IL‐8, IL‐6, IL‐1*β*, TNF‐*α*) elicited by LPS endotoxin and human immune cells that are necessary and sufficient to compromise intestinal barrier function.^[^ ^25^ ^]^ Interestingly, use of mechanically active Organ Chips also revealed that cessation of mechanical peristalsis‐like motions stimulates overgrowth of bacteria,^[^ ^26^, ^27^, ^28^ ^]^ as seen in patients with ileus and inflammatory bowel disease (IBD). Moreover, similar results were obtained using a human Colon Chip, which showed that peristalsis‐like mechanical deformations influence infection by the human pathogen *Shigella*.^[^ ^31^ ^]^ Finally, additional complexity has been integrated into Intestine Chip models by establishing a hypoxia gradient across the tissue–tissue interface, which enables extended co‐culture of human intestinal epithelium in direct contact (via its natural overlying mucus layer) with complex living human gut microbiome containing over 200 different types of microbial strains over multiple days in vitro.^[^ ^32^ ^]^ This is yet another advantage of human Organ Chips over static MPS cultures, organoids, and animal models, as they do not permit long‐term human microbiome co‐cultures.

Yet another example where Organ Chip experiments provide advantages over animal studies relates to modeling the blood–brain barrier, which has distinct permeability properties and transport functions in humans. Human Blood–Brain Barrier Chips have been developed that are lined by iPS cell‐derived human brain microvascular endothelium interfaced with either primary human brain pericytes and astrocytes^[^ ^33^ ^]^ or human iPS‐derived astrocytes and neurons,^[^ ^37^ ^]^ which reconstitute the high permeability restrictions of the human barrier. When differentiated appropriately, the iPS cell‐derived endothelium expresses high levels of human‐specific efflux pumps and tight junction proteins, which enables recapitulation of differential transport and selective transcytosis of antibodies and peptides, as well as extracellular vesicles, that have been observed in vivo.^[^ ^33^, ^34^, ^35^ ^]^ Blood–Brain Barrier Chips containing iPS‐derived human neurovascular cells isolated from patients also were able to recreate disease‐specific transporter deficiencies and barrier disruption.^[^ ^34^ ^]^ Moreover, when the vascular lumen was perfused with whole blood, the engineered blood–brain barrier protected neural cells from plasma‐induced toxicity and accurately predicted brain permeability of drugs. These observations raise the possibility that human Blood–Brain Barrier Chips may provide a more physiologically relevant way to develop shuttle molecules for delivery of therapeutic antibodies and other brain‐targeting therapeutics than mouse or non‐human primate models, which are used today.

**Figure 4:**
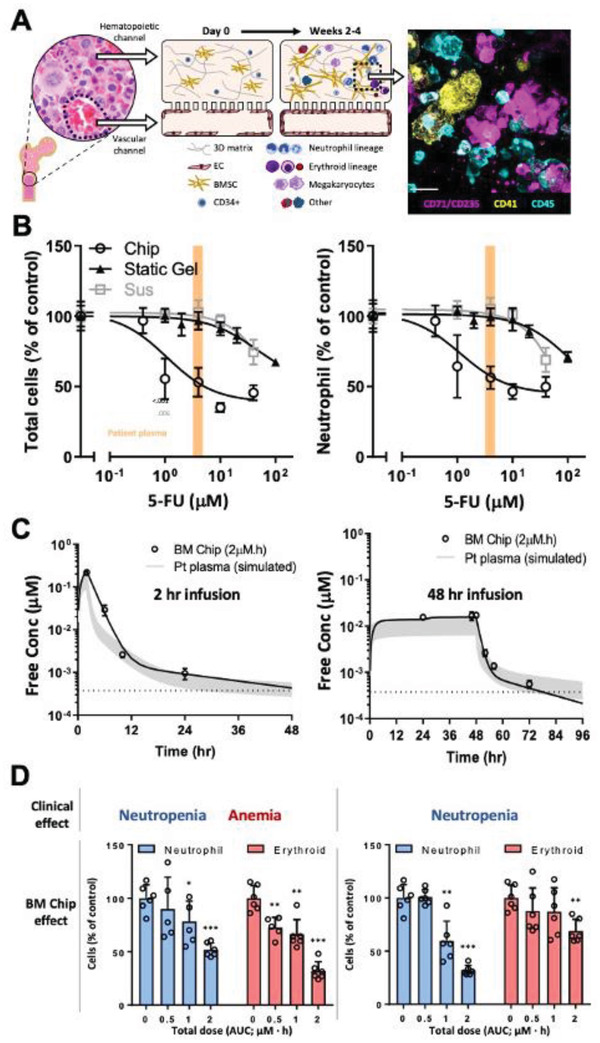
Prediction of clinically observed hematotoxicities at patient‐relevant drug exposures using a human Bone Marrow Chip. A) Left, schematic of bone and insert showing normal human marrow histology. Left middle, schematic of the human Bone Marrow Chip at the time of seeding, showing dispersed CD34+ progenitor cells and bone marrow stromal cells in an ECM gel filling the top channel, and a vascular endothelium beginning to line the adjacent channel. Right middle, within 2 weeks of culture, the endothelium covers all four sides of the lower channel, creating a vascular lumen, and the CD34+ cells undergo expansion and multilineage differentiation. Right, immunofluorescence view of a cross‐section through the ECM gel grown on‐chip for 14 days (yellow, megakaryocyte lineage; magenta, erythroid lineage; blue, neutrophil and other hematopoietic lineages; EC, endothelial cell). B) Effects of treating the Bone Marrow Chips, suspension cultures, and static ECM gel co‐cultures for 48 h with various doses of the cancer drug, 5‐fluorouracil (5‐FU) on total cell number (left) versus only neutrophil lineage cells (right)(***p < 0.001). The range of patient plasma 5‐FU concentrations for a 2 day infusion that are known to cause myelosuppression is indicated in orange. Note that the Bone Marrow Chip replicates this sensitivity to a clinically relevant dose exposure whereas the other in vitro models do not. C) Graphs showing that dynamic changes in concentrations of the cancer drug AZD2811 measured by mass spectrometry in chip outlet (circles), which were used to fit PK models of Bone Marrow Chip drug exposure (black line) for 2 and 48 h infusions. Note that these drug exposure profiles that were generated in the microfluidic Bone Marrow Chip closely resembled plasma levels of AZD2811 in vivo (grey), which were simulated for an average patient at a range of clinical doses based on the known PK characteristics of AZD2811. D) Graphs showing the effects of infusing the Bone Marrow Chips with varying doses of AZD2811 for 2 versus 48 h; total neutrophil (blue) and erythroid (red) cell numbers on day 12 are shown (***p < 0.001). Note that the clinical observation of unusual regimen‐specific neutropenia and anemia with 2 h dosing, but only neutropenia with 48 h dosing, was replicated on‐chip. Reproduced with permission.^[^ ^36^ ^]^ Copyright 2020, Springer Nature.

Different useful functionalities and biological mimicry has been demonstrated using a human Bone Marrow Chip that maintains differentiation and maturation of multiple blood cell lineages over 1 month by co‐culturing CD34^+^ cells and marrow‐derived stromal cells in a fibrin gel in one channel interfaced with human microvascular endothelium in the other (**Figure** **4**A).^[^ ^36^ ^]^ This chip reproduces marrow toxicities induced by exposures to clinically relevant doses of anti‐cancer drugs (Figure 4B) or ionizing radiation, as well as bone marrow recovery after drug‐induced myelosuppression, whereas other preclinical models do not. Importantly, using this microfluidic chip, it was possible to recapitulate both drug exposure profiles that were previously measured in PK studies in human patients (Figure 4C) and associated regimen‐specific myeloerythroid toxicity responses previously measured in those same patients (Figure 4D). Equally important, characteristic hematopoietic defects of a rare genetic disorder (Shwachman–Diamond syndrome) were recapitulated on‐chip by incorporating cells into the Bone Marrow Chips that were isolated from patients, and this work led to new insight in the underlying pathogenesis of these disease. The ability of this Bone Marrow Chip to replicate drug PK‐dependent effects, clinically relevant radiation sensitivities, and a rare human disease phenotype using patient‐specific cells under these dynamic modeling conditions, is a clear example of why human Organ Chips should be considered as alternatives to animal models.

Human Organ Chips also have been used to create “orthotopic” Cancer Chip models by growing tumor cells in organ‐relevant contexts, rather than relying on injecting human tumor cells into their organs of origin in mice. For example, when human non‐small‐cell lung cancer (NSCLC) cells that arise at the bronchiolar–alveolar interface but grow preferentially within alveoli in patients, were cultured in human Lung Alveolus and Airway Chips, they recapitulated these organ microenvironment‐specific behaviors by proliferating rapidly in the former and exhibiting tumor dormancy in the latter.^[^ ^37^ ^]^ In addition, these models replicated differences in responses to tyrosine kinase inhibitor (TKI) therapies observed in human patients, and revealed that breathing motions can influence lung cancer cell growth and invasion, as well as therapeutic responses. Again, these insights would not be possible using animal models.

In another cancer study, a microfluidic Organ Chip model of the human bone perivascular niche containing vascular cell networks and marrow‐derived mesenchymal stem cells within a native bone matrix with controlled flow hemodynamics and oxygen gradients revealed that human breast tumor cells exposed to interstitial flow persist in a dormant state on‐chip that is associated with increased drug resistance.^[^ ^38^ ^]^ A perfused model of metastatic tumor seeding in liver that incorporates human hepatocytes, nonparenchymal liver cells, and breast cancer cells, and which was instrumented with oxygen sensors and micropumps that enabled diurnal variations in nutrient and hormone profiles, was similarly used to recreate the human‐specific metastatic microenvironment and probe the paracrine effects between liver cells and metastatic tumor cells.^[^ ^39^ ^]^ More recently, a microfluidic model containing a 3D collagen gel populated by human liver cancer cells in one channel was challenged with human T cells engineered to express tumor‐specific T cell receptors in an adjacent channel while modulating oxygen levels and inflammatory cues to assess the preclinical efficacies of adoptive T cell immunotherapies in an immunosuppressive environment.^[^ ^40^ ^]^ These types of studies can provide insight into human‐specific cancer and immune cell responses that would not be possible with animal models.

Organ Chips with different fluidic designs have produced additional evidence showing that this technological approach can be used with primary or patient‐derived cells to gain insight into human‐relevant cellular and molecular mechanisms of pathogenesis, as well as drug actions, more effectively than animal models. For instance, a human Organ Chip model of the kidney proximal tubule was combined with quantitative systems pharmacology‐based computational models to enable in vitro‐to‐in vivo translation (IVIVT) of experimental results for the biomarker kidney injury molecule‐1 (KIM‐1), which predicted human drug‐induced renal toxicities, and helped to identify favorable dosing regimens.^[^ ^41^ ^]^ The same approach was leveraged to uncover a unique mechanism by which an FDA‐approved antibiotic drug (polymyxin) induces kidney injury, and to identify related chemical analogues that do not induce this toxicity.^[^ ^42^ ^]^ Another Kidney Chip was used to model viral infection of the kidney distal tubule and how it alters sodium reabsorption, which can lead to serum electrolyte abnormalities.^[^ ^43^ ^]^ Finally, a microfluidic model of the kidney glomerulus lined by human iPS cell‐derived kidney podocytes interfaced with kidney microvascular endothelium and exposed to cyclic mechanical deformations (mimicking changes in blood flow with each beat of the heart) reconstituted differential clearance of albumin and inulin via glomerular filtration, as well as albuminuria and podocyte injury induced by the cancer drug adriamycin.^[^ ^44^ ^]^

**Figure 5:**
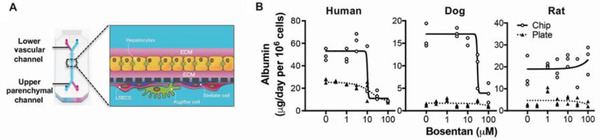
A human Liver Chip recapitulates species‐specific drug toxicities in rat, dog, and human. A) Schematic of the Liver Chip that contains an upper parenchymal channel lined by primary rat, dog, or human hepatocytes grown in ECM sandwich, while species‐specific liver sinusoidal endothelial cells (LSECs), Kupffer cells, and stellate cells are cultured on the opposite side of the same membrane in the lower vascular channel. B) Previously observed species‐specific effects of the drug bosentan on albumin secretion were recreated in microfluidic human, dog, and rat Liver Chips (Chip, open circles) whereas this was not replicated in static hepatocyte sandwich monocultures (Plate, closed triangles). Reproduced with permission.^[^ ^46^ ^]^ Copyright 2019, American Association for the Advancement of Science.

While most work in this field has focused on creating Organ Chips lined by human cells, species‐specific Organ Chips also have been made that, for example, can replicate the much higher susceptibility of humans to infection by enterohemorrhagic *Escherichia coli* (EHEC) compared to mice.^[^ ^45^ ^]^ Because fluids are continuously flowed through these chips, it was possible to carry out metabolomic analysis in this study using a Colon Chip, which led to the identification of specific chemical metabolites that are responsible for this different susceptibility to EHEC infection. Rat, dog, and human Liver Chips containing primary hepatocytes, liver sinusoidal endothelium, Kupffer cells and stellate cells also have been created that replicate species‐specific hepatotoxicities induced by multiple drugs (**Figure** **5**).^[^ ^46^ ^]^ This is an important finding because preclinical rat and dog liver toxicity studies are key requirements for regulatory approval, and they can often produce conflicting results, leading to confusion or removal of good drugs from the pipeline. These results suggest that human Organ Chips offer a potential way to cross‐validate these results between animals and humans early in the drug development process. These animal Organ Chips also might serve as replacements for animal validation studies, for example, in veterinary applications or be used to benchmark in vitro results versus findings obtained in past animal experiments.

More sophisticated microenvironmental control can be integrated into Organ Chips because of their microfluidic design. For example, continuous oxygen zonation can be generated in Liver Chips by regulating flow rate in the vascular and hepatic channels, which can provide insight into its contribution to human liver physiology, toxicology, and disease progression, as well as recruitment of circulating immune cells.^[^ ^47^ ^]^ By populating perfused Liver Chips with freshly cryopreserved hepatocytes from five different human donors, it also has been possible to quantify metabolic clearance rates for multiple drugs, and this led to the finding that albumin, urea, lactate dehydrogenase, and cytochrome P450 mRNA levels can serve as predictors of these pharmacologically relevant responses.^[^ ^48^ ^]^ In addition, by overlaying a computational, population‐level, physiologically based PK model, in vitro data from this study were related to their observed PK behavior in vivo, and model simulations predicted observed clinical concentration‐time profiles and associated population variability in drug metabolism. The same perfused Liver Chip also was able to recapitulate the clinical impact of an anti‐IL‐6R monoclonal antibody used to treat rheumatoid arthritis on small molecule drug PK, and it was shown to be mediated by modulation of cytochrome P450 enzyme activities.^[^ ^49^ ^]^

Another example where Organ Chips have been shown to be a valuable alternative to animals is in ophthalmologic drug development where results from animal experiments often do not translate to humans. Recently, a Retina Chip was created that is lined by retinal organoids derived from human iPS cells that form complex stratified retinal tissues containing more than seven different retinal cell types, and that permits physiological interactions between mature photoreceptor segments with retinal pigmented epithelium.^[^ ^50^ ^]^ This chip reproduced the retinopathic side effects of the anti‐malaria drug chloroquine and the antibiotic gentamicin in vitro, and now offers a preclinical human model to study drug efficacy and toxicities in a way that is not possible in animal models.

Perhaps the clearest argument against using animal models comes from studies that show human Organ Chips can replicate pathophysiological responses observed in humans that were never detected in preclinical animal studies. One example is a human Kidney Chip model of the proximal tubule that can replicate toxicities induced by cisplatin through a human‐specific Pgp efflux transporter that is not expressed in animals.^[^ ^51^ ^]^ Another is the demonstration that a human Blood Vessel Chip reproduces thrombotic toxicities in response to treatment with a therapeutic monoclonal antibody that caused deaths in human clinical trials even though this toxicity was not observed in preclinical animal studies.^[^ ^52^ ^]^ Finally, one challenge that is difficult for animal models is to faithfully mimic complex function of the human immune system, such as vaccination responses. However, recent studies suggest that this also may be possible using a human Organ Chip that supports self‐assembly of human blood‐derived B and T lymphocytes into germinal center‐like lymphoid follicles.^[^ ^53^ ^]^ Also, the human Bone Marrow Chip supports continued production of multiple blood cell lineages in vitro, as well as spontaneous intravasation of more differentiated blood cells into the endothelium‐lined vascular channel of these microfluidic chips,^[^ ^36^ ^]^ raising the possibility of creating fluidically linked single or multiple Organ Chip systems that incorporate all cells and tissues derived from the same patient, including circulating immune cells.

### 3.4 Multi‐Organ Chip Models

**Figure 6:**
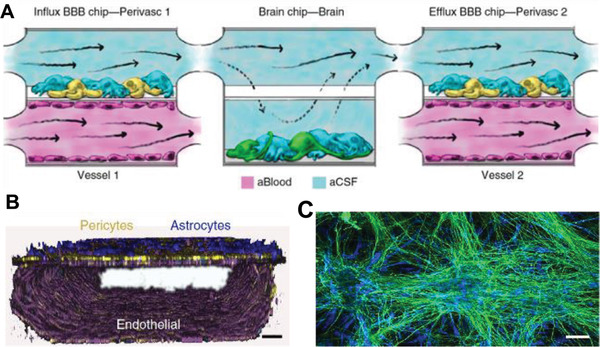
Multi‐Organ Chip model of the human neurovascular unit. A) Schematic of the influx Blood–Brain Barrier Chip containing brain microvascular endothelium (magenta) in its vascular channel separated by a porous membrane from brain astrocytes (blue) and pericytes (yellow) in the parenchymal channel through which medium mimicking cerebral spinal fluid (CSF) flows. The CSF fluid is transferred to a similar channel within a Brain Chip that is separated by a porous membrane from a channel containing cultured human brain neuronal cells (green) and astrocytes (blue), and from there to the parenchymal channel of a second efflux Blood–Brain Barrier Chip. Medium mimicking blood is flowed separately through the lower vascular channel. B) 3D confocal microscopic reconstruction of the Blood–Brain Barrier chip viewed from the side showing a continuous endothelium stained for VE‐cadherin (purple) forming a lumen in the lower vascular channel, as well as pericytes (F‐actin, yellow) and astrocytes (GFAP, blue) on the top surface of the porous membrane in the upper channel. C) Confocal fluorescence microscopic view of networks of human brain neurons (*β*‐III‐tubulin, green) and astrocytes (glial fibrillary astrocytic protein, GFAP, blue) in the lower compartment of the Brain Chip. Reproduced with permission.^[^ ^54^ ^]^ Copyright 2018, Springer Nature.

One of the greatest advantages of microfluidic Organ Chips over static engineered MPS and organoids is that multiple chips can be linked fluidically to model multi‐organ physiology and even create human “Body‐on‐Chips” models that can be used for PK studies. Two microfluidic human Blood Brain Barrier Chips (lined by primary brain microvascular endothelial cells interfaced with human astrocytes and pericytes) were fluidically coupled via their interstitial (cerebral spinal fluid) channels before and after a chip containing primary human brain neuronal networks (**Figure** **6**A–C) to model drug influx across the human blood–brain barrier, permeation into the brain parenchymal compartment, and efflux across the barrier.^[^ ^54^ ^]^ This model of the human neurovascular unit effectively mimicked the barrier opening properties of the psychoactive drug methamphetamine. It also permitted independent metabolomic analysis of each of the different fluidic compartments of the neurovascular unit, which revealed previously unknown metabolic coupling between the brain microvascular endothelium and neurons that would be impossible to do in animal studies.

In a separate study, a microfluidic multi‐organ model containing gut, liver, and circulating *T*_reg_ and Th17 cell components was used to model the contributions of microbiome and liver metabolism to IBD.^[^ ^55^ ^]^ This work revealed that microbiome‐derived short‐chain fatty acids can influence IBD severity based on the involvement of effector CD4 T cells. Multi‐Organ Chip systems also have been used to model preclinical drug toxicities and metabolic conversion,^[^ ^56^ ^]^ as well as sequential multi‐organ metabolism of drugs and xenobiotics that can impact therapeutic action and side effects.^[^ ^57^ ^]^ In the latter study, Organ Chips representing the jejunum, liver, and kidney (the major absorption, metabolism, and clearance organs) were evaluated, as well as skeletal muscle and neurovascular models, and organ‐specific ADME processing of various drugs was shown to be consistent with human clinical data.

It has even been possible to study organism‐level, hormonal cross‐talk, such as between insulin production in pancreas and glucose regulation in liver by fluidically coupling two microfluidic chips lined by human pancreatic islet microtissues and liver spheroids.^[^ ^58^ ^]^ A functional hormonal feedback loop between the human liver and insulin‐secreting islet tissues was demonstrated in this study, which suggests that it might be useful for studying diabetes in humans. Even more impressively, a functioning female reproductive system that can be used to study the human 28‐day menstrual cycle and pregnancy‐associated hormonal loops has been successfully recreated in vitro by fluidically coupling organ modules for the ovary, fallopian tube, uterus, cervix, and liver.^[^ ^59^ ^]^

**Figure 7:**
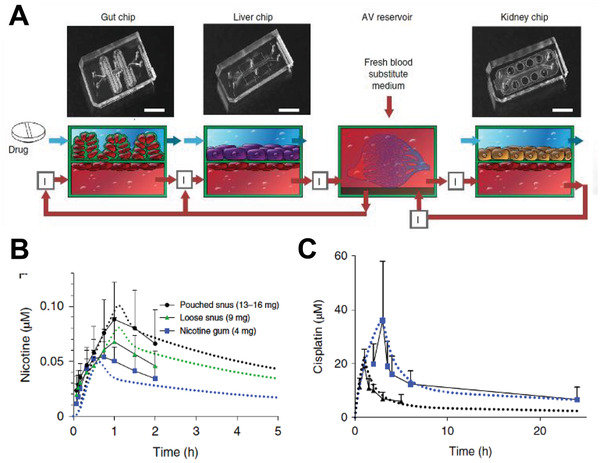
A first‐pass human Body‐on‐Chips model that predicts human PK parameters. A) Top, photographs of the Gut, Liver, and Kidney Chips that were fluidically coupled to each other and to an arterio‐venous mixer reservoir to create the first‐pass PK model in vitro. Bottom, diagrams of the different 2‐channel microfluidic chips with arrows indicating the manner in which the chips and reservoir are fluidically linked to each other and to the AV reservoir (red arrows indicate flow direction). B) Predictions of how nicotine blood concentrations will change over time for three different oral doses (different colored dashed lines) made by a physiological PK model using experimental data obtained from the human Body‐on‐Chips platform compared with previously published blood nicotine levels measured in patients who received orally administered nicotine in the form of nicotine gum (blue), pouched chewing tobacco known as “snus” (black), or loose snus (green) at three different doses (4, 9, and 13–16 mg). Note that the experimental platform was able to *quantitatively* predict these human PK behaviors. C) Graph showing changes in cisplatin concentrations in blood over time predicted by the same PK model using data from a Body‐on‐Chips configuration containing linked Liver, Kidney, and Bone Marrow Chips along with an arterio‐venous reservoir for infusion periods (dotted lines) of either 1 h (black) or 3 h (blue). Note that these predictions closely match previously published blood cisplatin levels measured in patients who received cisplatin injected intravenously over these same time periods (solid lines). Reproduced with permission.^[^ ^70^ ^]^ Copyright 2020, Springer Nature.

An outstanding example of a capability that Organ Chip technology offers that animal models do not relate to the modeling of human drug PK properties using human Body‐on‐Chips models that can maintain up to ten different human Organ Chips when fluidically coupled for 1 month in vitro.^[^ ^60^, ^61^ ^]^ PK studies have been carried out in many multi‐Organ Chip models by transfering fluids between them, and analyzing drug levels and metabolism using mass spectrometry.^[^ ^59^, ^62^, ^63^, ^64^, ^65^ ^]^ In some studies, qualitative predictions of drug toxicity responses have been made using PK and pharmacodynamic models with coupled Organ Chips.^[^ ^60^, ^66^, ^67^, ^68^, ^69^ ^]^ However, in these studies, the medium containing drug flowed directly from one parenchymal tissue type to another without passing through the endothelium, which is crucial for defining drug PK behavior in vivo, and thus, the physiological accuracy of these results is limited. However, more recently, it has been possible to quantitatively predict drug PK parameters (e.g., *C*_max_, *T*_1/2_) measured in human patients by applying physiologically based PK modeling to experimental data obtained with a human Body‐on‐Chips platform that coupled fluid flows between endothelium‐lined vascular channels of Liver, Kidney, and Intestine Chips to model first‐pass drug ADME behaviors (**Figure** **7**A).^[^ ^61^, ^70^ ^]^ This multi‐Organ Chip platform also incorporates an arterio‐venous fluid mixing reservoir so that drug concentrations within the vascular channels of all chips can be measured by sampling this shared compartment, much like taking venous blood samples in a patient. Quantitative PK predictions were demonstrated for two different drugs (cisplatin and nicotine) that were administered via two different routes (intravenous and oral, respectively) (Figure 7B), and predictions of cisplatin pharmacodynamics obtained in a fluidically linked Bone Marrow Chip also matched previously reported patient data.^[^ ^70^ ^]^ Together, these studies show how human Body‐on‐Chips approaches can enable quantitative IVIVT of human PK and pharmacodynamic parameters, as well as predict drug ADME properties and toxicities, which is not currently possible with animal models.

## 4 Advantages and Disadvantages of Organ Chip Models

From this analysis, it appears that human Organ Chips can provide much of the organ‐level information that Reviewer 3 is seeking when he or she requests additional animal validation studies, and even provide useful insight into multi‐organ physiology. The presence of dynamic fluid flow, tissue–tissue interfaces, and physiological mechanical cues enhances the fidelity of cell differentiation and increases expression of tissue‐specific functionalities on‐chip relative to conventional cultures, static MPS, and even organoids.^[^ ^14^, ^18^, ^23^, ^26^, ^27^, ^33^, ^36^, ^44^, ^46^, ^71^ ^]^ Tissues in Organ Chips also can be probed using virtually any type of analytic technique that is used in other in vitro models or animal studies, including methods leveraging high resolution microscopy, flow cytometry, transcriptomics, proteomics, metabolomics, and histological analysis, and as recently demonstrated, automated high content confocal imaging with fluorescent reporters.^[^ ^72^ ^]^ In addition, because Organ Chips can be instrumented with in‐line electrical, chemical, mechanical, and optical sensors (both alone and in combination), they can incorporate real‐time readouts and enable probes of critical cell and tissue functions, including tissue barrier integrity, neuronal cell electrical activity, oxygen utilization, pH, molecular transport, and multiple other relevant measures of differentiation state and viability.^[^ ^10^, ^11^, ^32^, ^47^, ^73^, ^74^, ^75^, ^76^, ^77^, ^78^, ^79^ ^]^ The ability to control the contents of different parallel flow paths individually enables regulation of oxygen and chemical gradients across tissue–tissue interfaces as well, which permits stable co‐culture of living human tissues in direct contact with living complex microbiome as in the human intestine,^[^ ^32^ ^]^ as well as recreation of oxygen zonation patterns in organs, such as the liver.^[^ ^47^ ^]^ All of these measurements can be carried out under dynamic flow conditions as occurs in vivo, and many would be difficult, if not impossible, to carry out in animal studies or even in human trials.

Organ Chips offer a potentially useful alternative to animal testing, but they cannot replicate many complex functions and responses that are still better studied in animals. The most obvious examples relate to studies focused on cognitive functions and complex organismal behaviors, which remain beyond the capabilities of models that rely on cultured cells. Similarly, while brain neurons have been integrated into some microfluidic Organ Chips,^[^ ^34^, ^54^ ^]^ peripheral neurons have not; this is an area that clearly needs greater exploration before animal studies can be replaced. Quantitative analysis of the effects of large‐scale forces and deformations on large organs (e.g., long bones, spinal disks, whole joints), or of the contributions of macroscale 3D architecture to host responses (e.g., infections of the lung apex vs base), also cannot be studied using Organ Chip technology, although some simplified models can be developed to study some of the underlying cellular mechanisms (e.g., local effects of compression on bone or cartilage tissue). While mimicry of some hormonal axes has been accomplished using microfluidic Organ Chips, such as the reproduction of the menstrual cycle in vitro,^[^ ^58^ ^]^ it is difficult to model temporal variations in many different human hormones simultaneously as can occur in vivo; moreover, many bloodborne regulatory factors that could be important for biological regulation are still unknown. But because of flow control, it is possible to replicate temporal changes in hormone levels in Organ Chips for hormones that have been well characterized in vivo.

The lack of fat tissues that can absorb many drugs and lipophilic compounds in vivo also could produce abnormal results in experiments studying how drugs distribute in multi‐Organ Chip models. Some materials that are used to construct Organ Chip devices, such as PDMS, can absorb lipophilic drugs and hence, this could similarly interfere with drug studies; interestingly, however, by quantifying the level of absorption into these materials (e.g., using mass spectrometry), this feature can be used to mimic fat absorption in human Body‐on‐Chips modeling.^[^ ^61^, ^70^ ^]^ Another advantage human Organ Chips could potentially provide relative to animal models is the ability to replicate human‐specific immune and inflammatory responses that are not seen in other species. Circulating immune cells have been incorporated into multiple human Organ Chip models^[^ ^9^, ^14^, ^18^, ^21^, ^25^ ^]^ and complex vaccination responses have been replicated on‐chip.^[^ ^53^ ^]^ But to meet this goal of fully recapitulating human immune responses in vitro, it will be necessary to incorporate tissue‐resident immune cells and obtain parenchymal, connective tissue, endothelial, and immune cells all from the same donor.

Another challenge that the Organ Chip field faces relates to cell sourcing. While established cell lines generally fail to replicate tissue‐specific functions with high fidelity, primary human cells and organoids can display significant donor‐to‐donor variability. The quality of commercially available primary cells (e.g., doubling time, passage number, differentiation potential) also can vary greatly between different suppliers and even between different lots from the same company. However, these issues can be managed by establishing protocols for “qualifying” each cell source in terms of the consistency and quality of their growth and differentiation potential before initiating critical experiments. Moreover, inter‐donor variability, while frustrating relative to working with established cell lines or inbred animals, is much more clinically relevant as it mimics the diversity of responses seen in clinical trials and broader human populations. This challenge can be overcome by repeating studies with cells from multiple donors, and exploring whether male and female cell sources produce different results can lead to helpful insights as well. Interestingly, however, studies with Lung Airway Chips^[^ ^18^, ^19^ ^]^ and Bone Marrow Chips^[^ ^36^ ^]^ suggest that results can be quite reproducible between different donors.

Finally, it is important to note that Organ Chips do not mimic the function of a whole organ; rather, they are effectively living 3D cross‐sections of a major functional subunit of an organ, such an alveolus, airway, glomerulus, or proximal tubule. So before any animal model can be replaced, the Organ Chip must be designed so that it contains the key features of the relevant organ unit that is the key site of this activity, while minimizing system complexity. For example, while a Lung Alveolus Chip containing only alveolar epithelium, endothelium, and immune cells might be used to replace an animal model of pulmonary edema that is focused on preventing pulmonary vascular leakage, it would need to be modified by adding additional connective tissue cells to replace an in vivo pulmonary fibrosis model. Thus, the goal is not to use Organ Chips to replace animal models in a generic way; rather, it is to replace one specific type of animal model at a time. Similarly, human Body‐on‐Chips platforms need to be designed to model the organism‐level properties of interest, and recent studies suggest that meaningful results can be obtained using this approach that may be even more useful than those obtained from animal studies, as demonstrated by the ability to quantitative predict human drug PK parameters using a first pass intestine–liver–kidney model,^[^ ^70^ ^]^ which could potentially accelerate clinical trials. This inability of a single Organ Chip to model all facets of that organ's function, and the need to modify both single and multiple Organ Chip systems based on the questions being posed, are challenges that animal models do not have. Furthermore, regardless of the device design, all chips must be experimentally validated in terms of their ability to recapitulate the key physiological or pathophysiological properties of the particular organ, disease state, or multi‐organ coupling that is the focus of the study, and to do it in a consistent and robust manner.

## 5 Conclusion

This article began with a simple question: does it make sense for reviewers to demand animal validation studies before being willing to fund grants or share articles with the larger scientific community, and if not, is there a better alternative? As described above, the problem mainly lies in the fact that many animal models are physiologically irrelevant when considering human disease, and thus, demanding use of a poor animal model for the sole sake of “satisfying Reviewers” should be discouraged. Given recent advances in human Organ Chip technologies, I suggest that these new forms of human experimentation in vitro provide more physiologically and clinically relevant preclinical models for studying both pathophysiology and pharmacological responses than many animal studies, and they should be considered for this purpose in the future. Organoids and other MPS models that often can be carried out at a higher throughput than microfluidic Organ Chips also have a very important role to play, particularly in studies focused on cellular and molecular mechanisms of tissue regulation and drug action. Organ Chips can be used for this purpose as well; however, their greatest novelty comes from their ability to reconstitute dynamic vascular perfusion, tissue–tissue interfaces, and organ‐specific mechanical cues that are important for in vivo‐like functions, and to provide an ability to selectively add complexity to these models in the form of soluble factors and different cell types (e.g., epithelial, endothelial, connective tissue, immune, nerve, muscle, commensal microbes). Thus, Organ Chips are a form of synthetic biology that allows one to build artificial models with defined composition at the cell, tissue, and organ level, and thereby, to identify key cellular and molecular contributors to human physiology and pathophysiology. For example, it would be extremely difficult, if not impossible, to identify the individual contributions of microbiome, mechanical cues, and specific cytokines to pathophysiology at the organ and multi‐organ level using animal models, whereas this has been easily done using Organ Chips. At the very least, manuscript and grant reviewers should first consider non‐animal alternatives, such as human Organ Chips, and second, whether or not a fully validated animal model is available.

Importantly, this article is *not* an argument to stop all animal testing, and to carry out all validation studies using human Organ Chips instead. Clearly, we will need to continue some forms of animal testing for many years to come in order to cross‐validate Organ Chip results with the current state‐of‐the‐art, and to progressively convince animal researchers and regulatory scientists of their value. And it is likely that some animal studies will be very hard to replace (e.g., those focused on cognition and behavior). Rather, my goal here is to initiate a conversation among members of our community, and to ask scientists who serve as reviewers to consider the realities as they stand today. As our collective interest is in human health, our goal should not be to validate results against animals, but rather against humans, because in the end we all know that mice are not men. Perhaps it is time for reviewers to accept this reality.

## Conflict of Interest

The author has the following potential conflicts: Emulate Inc., equity, consulting, chair of SAB; BOA Biomedical Inc., equity, consulting, chair of SAB, board member; Free Flow Medical Device, equity; SynDevRx, equity; Consortia Rx, equity, board member; Roche, consulting; Astrazeneca, sponsored research; Fulcrum Therapeutics, sponsored research; Kraft Heinz, sponsored research; and inventor of multiple patent applications.
